# Validation of Global Self-Rated Health and Happiness Measures Among Older People in the Yilan Study, Taiwan

**DOI:** 10.3389/fpubh.2020.00346

**Published:** 2020-07-31

**Authors:** Yen-Huai Lin, Hsi-Chung Chen, Nai-Wei Hsu, Pesus Chou

**Affiliations:** ^1^Department of Medical Imaging, Cheng Hsin General Hospital, Taipei, Taiwan; ^2^Community Medicine Research Center, National Yang-Ming University, Taipei, Taiwan; ^3^Department of Psychiatry and Center of Sleep Disorders, National Taiwan University Hospital, Taipei, Taiwan; ^4^Division of Cardiology, Department of Internal Medicine, National Yang-Ming University Hospital, Yilan, Taiwan; ^5^Department of Medicine, School of Medicine, National Yang-Ming University, Taipei, Taiwan; ^6^Public Health Bureau, Yilan, Taiwan

**Keywords:** health-related quality of life, older adults, self-rated health, self-rated happiness, validation, Yilan study

## Abstract

**Background:** Single-item measures of physical and mental health are feasible for older adults, but their validity for that age group is unclear. This study tested validity of a global self-rated health and a global self-rated happiness measure in a large sample of community-dwelling older adults in Taiwan.

**Methods:** A cross-sectional sample of 3,982 men and women aged 65 or older in Yilan, Taiwan, provided data on global self-rated health and happiness using 100-point numerical scales. The Physical Component Summary of the 12-Item Short Form Health Survey (version 2) and the Groningen Activity Restriction Scale were used to test the validity of the self-rated health item. The Mental Component of that 12-item scale and the Hospital Anxiety and Depression Scale were validators regarding the self-rated happiness item. Criterion validity was tested using the 12-Item Short Form Health Survey (version 2).

**Results:** The correlations between the self-rated health and happiness measures and the 12-Item Short Form Health Survey (version 2) validators were positive and statistically significant, supporting convergent validity. Sufficient divergent validity was demonstrated through the negative and significant relationship between the self-rated health item and the Groningen Activity Restriction Scale scores and the negative and significant relationship between the self-rated happiness item and the Hospital Anxiety and Depression Scale. Optimal cut-off scores for physical and mental health states depended on age and gender.

**Conclusion:** The global self-rated health and happiness measures were validated. Cut-off scores for evaluating older adults' physical and mental health should be age- and gender-specific.

## Introduction

Population aging is dramatically increasing around the world, and evaluating and eliminating health problems for older adults in the community are important aspects of public health. Physical and mental health assessments are major components of comprehensive geriatric assessments (1). For these evaluations, the paper-and-pencil questionnaire remains the most feasible tool, but older adults' cognitive, functional and aging-related vision and hearing losses tend to increase the costs, decrease the validity and reduce older people's willingness to participate in these evaluations. Therefore, valid, simple, and easy ways to measure physical and mental health are important to community-based geriatric medicine. Asking a general or global question has the advantage of being a relatively low-cost way to easily collect, score and interpret these valuable data.

Global self-rated health measures have been widely applied in general populations, developing countries, militaries, and many societies, and they have been found to adequately and objectively indicate physical health, mental health, chronic diseases, unhealthy behaviors and physical functioning (2–5). Despite the potential value of global measures, they are not widely used for older adults, partly because their validity has not been established for that age group. It is suspected that global self-ratings might not be sensitive enough to distinguish among subtle individual differences because of older adults' declining physical functionality. However, most of the global self-rated health measures' response options are ordinal scales, which tend to be relatively insensitive to skewed distributions and might compromise validity (6). It has been suggested that a numerical rating scale in response to a global question might be more accurate for measuring older adults' health status (7). To the best of our knowledge, no previous study has investigated the validity of global self-rated health measures with numerical scales for older adults in Asia.

Regarding mental health, global self-rated happiness measures have been developed. Happiness is a combination of positive hedonic, cognitive, and affective states, and individual assessments of personal happiness are influenced by individual, cultural and societal factors (8). Similar to self-rated physical health, the instruments use ordinal response scales (9–11). Moreover, although comprehensive verifications of construct validity should evaluate divergent as well as convergent validity, most global self-rated happiness measures have been assessed for convergent validity using constructs that measure emotions or attitudes, such as life satisfaction (12), or happiness (13). The 12-Item Short Form Health Survey (version 2) [SF-12v2] is a multi-dimensional instrument that comprises physical, mental, emotional, and social health dimensions to evaluate health-related quality of life, which is reasonable as an external validator for self-rated health and happiness. Although the physical component summary (PCS) of the SF-12v2 has previously been used to validate global self-rated health (14), no previous study has used its mental component summary (MCS) as an external validator of global self-rated happiness. Besides, we know of just one multi-item self-rated happiness scale of which the divergent validity was assessed using depression and anxiety (15). Further, similar to measures of self-rated health, no global self-rated happiness measure with numerical scales has been assessed regarding its construct validity in older adults.

Therefore, the present study investigated validity of a global self-rated health measure and of a global self-rated happiness measure. The sample was a large cohort of community-dwelling older adults. A comprehensive set of external validators, including SF-12v2 was used to evaluate construct validity and the criterion validity of the two self-rated measures.

## Methods

### Participants

The data used for this study were derived from the Yilan Study, a community health survey conducted by the Community Medicine Research Center of National Yang-Ming University and National Yang-Ming University Hospital in Taiwan. The data were collected between January 2012 and November 2016. The household registration lists were protected under the personal data protection law of Taiwan and, therefore, a sample was randomly selected from all city residents aged 65 years or older living in Yilan City. Trained interviewers went to the participants' homes for face-to-face interviews. The final sample comprised 3,982 individuals. The details of the sampling methods have been previously reported (16, 17). The institutional review board of National Yang-Ming University Hospital (IRB No. 2011A016) approved the study. Informed written consent was obtained from all the participants, and all methods were performed according to the relevant guidelines and regulations.

### Instruments

#### Verifying Convergent and Divergent Validity of the Global Self-Rated Health Measure

The participants were asked to self-rate their general health status on a scale of zero to 100 where higher scores indicated better health. They answered the following question: “How would you rate your present health status?” The PCS of the SF-12v2 was used to assess the convergent validity of the global self-rated health measure. The Chinese translation of the SF-12v2 previously was found to be a valid instrument (18). The Groningen Activity Restriction Scale (GARS) was used to assess the divergent validity of the global self-rated health measure. The GARS is considered a valid measure for assessing disability in activities of daily living (ADLs) and instrumental activities of daily living (IADLs) in older people (19).

#### Verifying Convergent and Divergent Validity of the Global Self-Rated Happiness Measure

The participants evaluated their happiness by responding to the question: “In general, how would you rate your current state of happiness?” They rated themselves on a scale of zero to 100 and higher scores indicated more happiness. To assess the convergent validity of global self-rated happiness, the Mental Component Summary (MCS) of the SF-12v2 was used. The Chinese translation of this part of the SF-12v2 is considered valid (18). The Hospital Anxiety and Depression Scale (HADS) was used to determine the divergent validity of the global self-rated health measure. The HADS is a reliable instrument used to measure clinical and subclinical anxiety and depression in the general population (20, 21), and the Chinese translation of the HADS is considered valid (22).

#### Criterion Validity of the Global Self-Rated Health and Happiness Measures

Previous studies that investigated the cut-off scores for PCS or MCS for predicting physical or mental health outcomes (23–25) implied the inclusiveness of the SF-12v2 regarding overall health status. Thus, the two components' scores were effective options for assessing optimal physical and mental health scores with respect to global questions. A previous study has used the SF-12v2 as a validation instrument for global self-rated health (14). The PCS and the MCS use norm-based scoring in which scores higher (or lower) than 50 indicate better (or worse) physical (or mental) health relative to that of a given sample's population (26). In addition, previous studies have found that cut-off values on the PCS and MCS below 50 points were related to poor physical and mental health, respectively (23–25, 27). Accordingly, the present study used a score of 50 or higher on the PCS and MCS as the cut-off scores to indicate the optimal self-rated health and happiness scores in the self-rated measures, respectively. Regarding criterion validity, the receiver operating characteristics (ROC) curve was used to determine the cut-off scores on global self-rated health and happiness based on a score of 50 in the PCS and MCS.

### Statistical Analysis

The Chi-Square goodness-of-fit test was used to compare the demographic characteristics of the sample to those of the Yilan city population. Pearson's correlation coefficients were calculated to investigate the relationships among the two global measures, PCS, MCS, GARS, and HADS. The size of the correlation is defined as high, moderate, and low by scores of: 0.70–0.89, 0.40–0.69, and 0.10–0.39, respectively (28). A stepwise multivariable linear regression analysis estimated the associations between the PCS and self-rated health and between the MCS and self-rated happiness. A general linear model was used to compare the between-group differences in self-rated health and in self-rated happiness with and without controlling for the effects of gender and age. The Youden's index was calculated from the ROC curve to determine the optimal cut-off scores for self-rated health and happiness based on cut-off scores of 50 on the PCS and MCS, respectively. All statistical tests were two-tailed, and *p* < 0.05 was considered statistically significant. The statistical software package SPSS for Windows, Version 19.0 (SPSS Inc., Chicago, IL, USA) was used to perform all the analyses.

## Results

### Sample Characteristics

Table 1 presents the sample's demographic characteristics. About 57% of the sample was female, and compared to the registered residents of Yilan who were aged ≥ 65 in 2012 (29), the sample was significantly older (χ^2^ = 99.2, df = 1, *p* < 0.001) and more likely to be female (χ^2^ = 21.1, df = 1, *p* < 0.001). The PCS mean was 46.7, and the MCS mean was 57.9. The self-rated health mean was 69, and the self-rated happiness mean was 74.

**Table 1:** Descriptive statistics (*n* = 3,982).

**Variable**	**Number of cases *(n)***	**Percentage (%)**	**Mean**	**Standard deviation**
Age (in years)				
65–74	1,841	46.2		
75 or older	2,141	53.8		
Gender				
Male	1,711	43.0		
Female	2,271	57.0		
Self-rated measures				
Self-rated health (range: 0–100)			69.0	12.5
Self-rated happiness (range: 0–100)			74.0	14.5
Short Form-12v2				
Physical Component Summary (PCS) (range: 11.6–71.1)			46.7	10.0
Mental Component Summary (MCS) (range: 10.4–77.8)			57.9	8.3
Groningen activity restriction scale (GARS) (range: 18–72)			23.7	12.3
Hospital Anxiety And Depression Scale (HADS) (range: 0–36)			4.6	5.0

### Convergent and Divergent Validity

Table 2 shows the bivariate correlation coefficients among the self-rated health, self-rated happiness, PCS, MCS, GARS, and HADS variables. Regarding global self-rated health, the convergent validity was tested by the correlation between global self-rated health and PCS (*r* = 0.471, *p* < 0.001) and divergent validity was indicated by the correlation between global self-rated health and GARS (*r* = −0.316, *p* < 0.001). Regarding global self-rated happiness, the correlation between global self-rated happiness and MCS assessed convergent validity (*r* = 0.357, *p* < 0.001) and the correlation between global self-rated health and HADS assessed divergent validity (*r* = −0.423, *p* < 0.001). The overall sizes of correlations between global self-rated health and happiness with external validators were low to moderate. In addition, the correlation between self-rated health and self-rated happiness was high (*r* = 0.600, *p* < 0.001).

**Table 2:** Bivariate correlation matrix among the six measures of health and happiness^a^ (*n* = 3,982).

**Measure**	**1**	**2**	**3**	**4**	**5**	**6**
1. PCS	1					
2. MCS	0.038^*^	1				
3. HADS	−0.264^***^	−0.583^***^	1			
4. GARS	−0.766^***^	−0.355^***^	0.319^***^	1		
5. Global self-rated health	0.471^***^	0.249^***^	−0.318^***^	−0.316^***^	1	
6. Global self-rated happiness	0.310^***^	0.357^***^	−0.423^***^	−0.264^***^	0.600^***^	1

* *p < 0.05*,

*** *p < 0.001*.

a *PCS, physical component summary; MCS, mental component summary; HADS, hospital anxiety and depression scale; GARS, groningen activity restriction scale*.

To examine the relationships between the PCS and the MCS and the two global self-rated measures, stepwise multivariable linear regression analysis was performed. The goal was to determine the strengths of the associations. Table 3 shows the results. Model 1 shows that, net of the effects of age and gender, global self-rated health was related to PCS (*R*^2^ = 0.221). With every unit increase in the global self-rated health, the PCS increased by 0.351. In Model 2, global self-rated health and global self-rated happiness related to MCS, and the relationship of global self-rated happiness was stronger than that of global self-rated health (*R*^2^ = 0.127). With every unit increase in the global self-rated happiness, the MCS increased by 0.179.

**Table 3:** Stepwise multivariable linear regressions for the associations of global self-rated health and happiness with the Physical Component Summary (PCS, Model 1^a^) and Mental Component Summary (MCS, Model 2^a^).

**Model 1: PCS**				**Model 2: MCS**			
**Variable**	**B**	**95% CI**	**Cum *R*^**2**^**	**Variable**	**B**	**95% CI**	**Cum *R*^**2**^**
Global self-rated health	0.351	0.330, 0.373	0.221	Global self-rated happiness	0.179	0.159, 0.200	0.127
Gender (ref.: female)				Gender (ref.: female)			
Male	0.564	0.027, 1.101	0.248	Male	0.917	0.434, 1.400	0.130
Age (in years; ref.: 75+)				Age (in years; ref.: 75+)			
65–74	3.128	2.596, 3.661	0.247	65–74 years	0.555	0.076, 1.034	0.133
Global self-rated happiness	–	–	–	Global self-rated health	0.029	0.005, 0.053	0.131

a *Final models are shown; unstandardized coefficients (B), 95% Confidence Intervals (CI), and cumulative R^2^ (Cum R^2^)*.

### Criterion Validity

The mean global self-rated health scores were significantly different by age and gender among the participants with PCS scores of 50 or higher (Table 4). Controlling for gender differences, the mean PCS score was still significantly higher among those aged 75 years or older compared to the younger participants. Among the participants with MCS scores of 50 or higher, the mean global self-rated happiness score was significantly different by age (those younger than 75 had a higher mean score) and gender (the males' mean was higher than the females' mean) even after controlling for the effects of gender or age (Table 4). Therefore, because of the gender and age differences, we calculated optimal scores for global self-rated health and global self-rated happiness separately by age and gender.

**Table 4:** Mean differences in global self-rated health and global self-rated happiness among the participants with PCS scores at or above the mid-score and MCS scores at or above the mid-score by age group and gender^a^.

**Variable**	**Global self-rated health**						**Global self-rated happiness**					
	**PCS ≥ 50**			**MCS ≥ 50**			**PCS ≥ 50**			**MCS ≥ 50**		
	**Mean**	**SD**	***p***	**Mean**	**SD**	***p***	**Mean**	**SD**	***p***	**Mean**	**SD**	***p***
Age												
65–74	73.6	10.7	0.018	70.6	11.6	0.015	78.0	13.4	0.966	76.7	13.4	<0.001
75+	74.7	10.8		69.6	12.4		78.0	12.2		74.8	13.1	
Gender												
Male	74.6	10.8	0.04	70.9	12.0	0.001	78.1	12.5	0.549	76.2	13.0	0.076
Female	73.6	10.7		69.5	12.0		77.8	13.1		75.3	13.5	
	**EMM**	**95% CI**	***p***	**EMM**	**95% CI**	***p***	**EMM**	**95% CI**	***p***	**EMM**	**95% CI**	***p***
Age^b^												
65–74	73.7	73.0, 74.3	0.034	70.8	70.1, 71.4	0.006	78.0	77.2, 78.8	0.970	76.8	76.2, 77.5	<0.001
75+	74.7	74.0, 75.4		69.6	69.0, 70.2		78.0	77.1, 78.8		74.8	74.2, 75.4	
Gender^c^												
Male	74.6	73.9, 75.3	0.086	70.9	70.3, 71.6	<0.001	78.1	77.3, 79.0	0.567	76.3	75.7, 77.0	0.026
Female	73.8	73.1, 74.4		69.4	68.9, 70.0		77.8	77.0, 78.6		75.3	74.7, 75.9	

a *PCS, physical component summary; MCS, mental component summary; EMM, estimated marginal mean*.

b *Estimates are gender adjusted*.

c *Estimates are age adjusted*.

**Figure 1:**
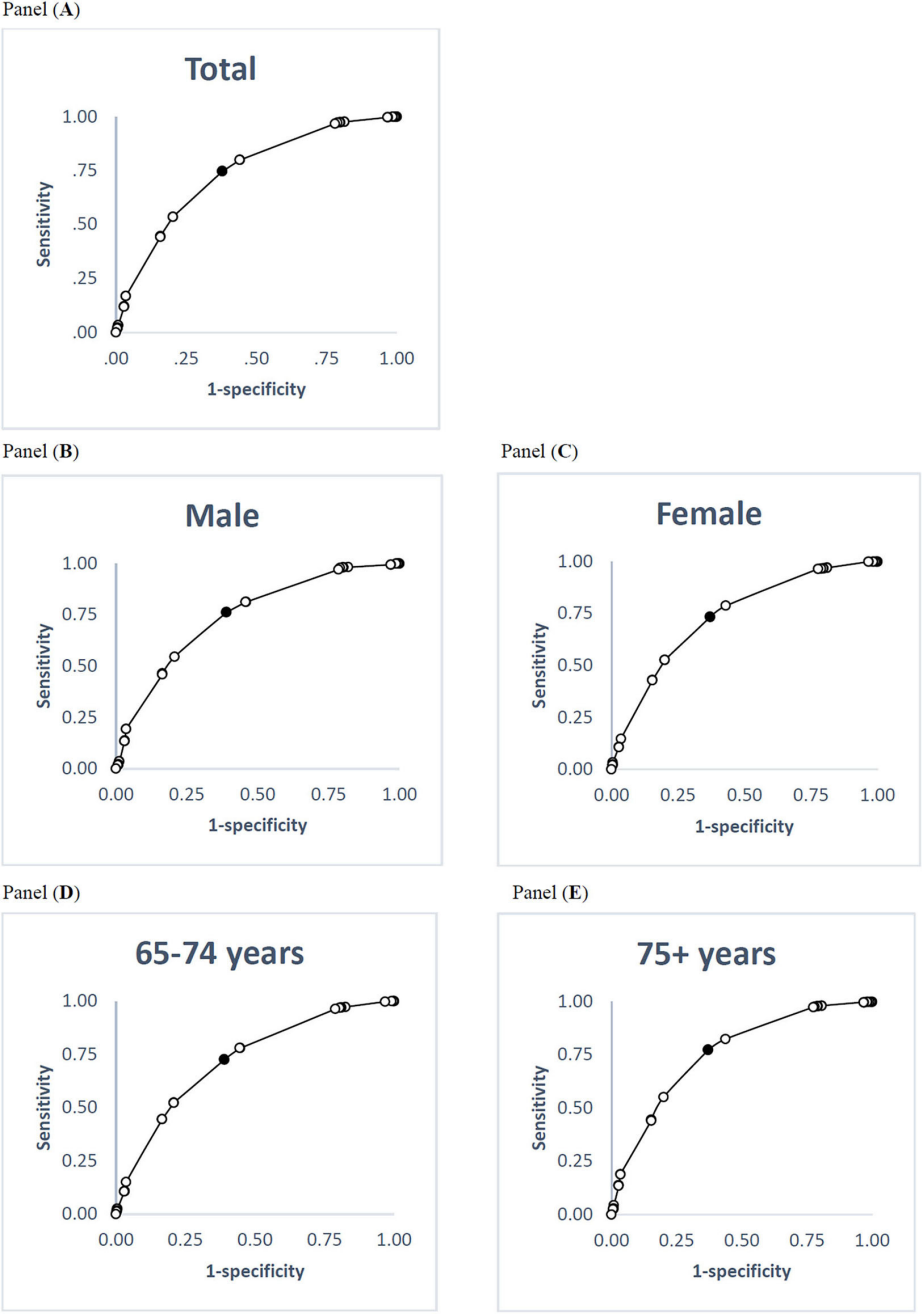
ROC curves for global self-rated health relative to the PCS cut-off scores of 50. **(A)** Total participants; **(B)** male; **(C)** female; **(D)** 65–74 years; **(E)** 75+ years. Black point indicates the optimal cut-off point. ROC, receiver operating characteristics; PCS, physical component summary.

**Figure 2:**
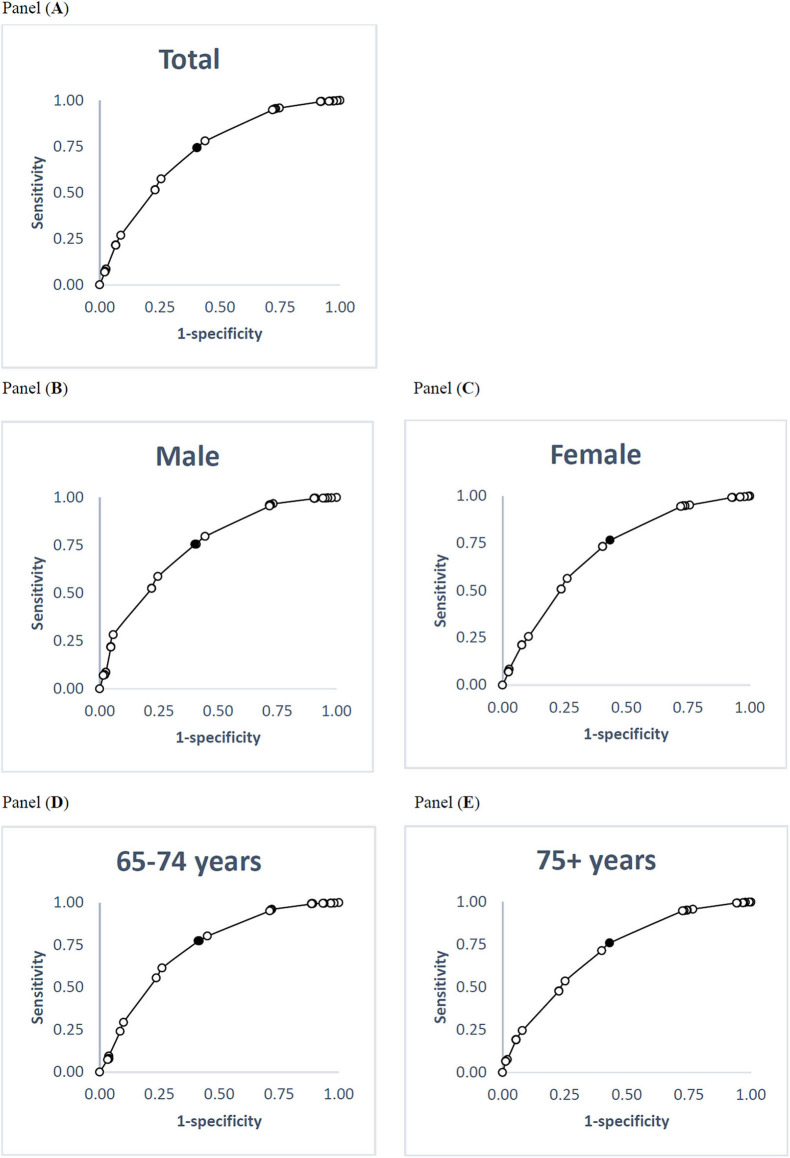
ROC curves for global self-rated happiness relative to the MCS cut-off scores of 50. **(A)** Total participants; **(B)** male; **(C)** female; **(D)** 65–74 years; **(E)** 75+ years. Black point indicates the optimal cut-off point. ROC, receiver operating characteristics; MCS, mental component summary.

At a score of 50 on the PCS, the global self-rated health measure's cut-off scores were calculated as 68.5 overall, 68.5 for males, 67.0 for females, 67.0 for those aged 65–74 years, and 69.0 for those aged 75 or older (Table 5). At a score of 50 on the MCS, the cut-off scores on the global self-rated happiness measure were calculated as 69.5 overall, 69.5 for males, 62.5 for females, 69.5 for those younger than 75 years, and 62.5 for those aged 75 years or older (Table 5). Figures 1, 2 illustrate ROC curves among total participants and subgroups. The sensitivity, specificity and area under curve are shown in the Table S1.

**Table 5:** Optimal cut-off scores on global self-rated health and global self-rated happiness when the PCS and MCS cut-offs are scores of ‘50'; n = 3,982.

**Group**	**Physical component summary (PCS)**	**Mental component summary (MCS)**
	**Optimal cut-off scores on global self-rated health**	**Optimal cut-off scores on global self-rated happiness**
Total sample	68.5	69.5
Gender		
Male	68.5	69.5
Female	67.0	62.5
Age (in years)		
65–74	67.0	69.5
75+	69.0	62.5

## Discussion

Using a large number of elderly participants in Taiwan and various measurements as external validators, this study verified global measures of self-rated health and happiness, which differentially represent physical and mental dimensions of elders' health, respectively. In addition, cut-off scores for evaluating elderly adults' physical and mental health should be determined depending on age and gender.

Although the present study found global self-rated health and happiness were correlated with each other, the global self-rated health measure had a stronger relationship to the PCS, supporting the results of a previous study (14). That previous study also examined the relationship between global self-rated health and MCS (14). Although the present study found that global self-rated health significantly related to MCS, it was not a stronger association than that of global self-rated happiness with MCS. Our findings suggest that the PCS was a relatively better validator of global self-rated health and MCS was a relatively better validator of global self-rated happiness.

Gender related to the measures of self-rated health and happiness. Many previous studies have found that women rate their health lower than men rate their health (30–32). We found a similar gender difference, which might be because men generally compare their health to other men, whereas women tend to rate their health based on their family members' opinions (33). However, the gender difference regarding global self-rated happiness was inconsistent with previous results. One previous study found that women reported higher happiness than men in the past, but men had higher happiness than women at present (34). We found that men had higher self-rated happiness than women. Therefore, it is important to understand gender differences in self-rated health and happiness among older adults.

Self-rated health is believed to decrease with age (35, 36), but whether the influence of age continues into old age is unclear. Several previous studies found better self-rated health among older than younger old people (37, 38). Our results support these findings. A previous study on age-related changes in self-rated health among older men considered age, time and cohort effects and found that self-rated health was influenced by time, but there were no age or cohort effects (39). The researchers explained the absence of an age effect first by evoking the reference-group hypothesis, which contends that, among older people who perceive poor health and disability as their age-related norm, those who are relatively healthy rate their health positively. Another explanation was the health survivor effect, which proposes that people who do not have serious health problems are more likely to survive to older ages, so their assessments are objectively accurate.

Age also has been positively associated with happiness, and older people have been found to self-rated happiness higher than younger people (40, 41). The socio-emotional selectivity theory proposes that older people accumulate emotional wisdom that helps them to select emotionally satisfying activities and experiences (40); however, similar to self-rated health, it is not clear whether the influence of age on self-rated happiness continues into old age. Indeed, the age-happiness relationship among older people is often not discussed (40, 41). However, a recent study reported that happiness declined among very old people in Europe (42).

We found that the participants aged 75 or older with MCS scores of 50 or higher were less happy than their younger counterparts, which contradicts the socio-emotional selectivity theory. One previous study reported that older Chinese people had a high prevalence of mental disorders (43), which might contribute to low self-rated happiness. Moreover, our sample was drawn from the population of Yilan, which is an agricultural suburb. If they compared themselves to younger old people, the older old people in our sample might have thought they had fewer resources and less social support and rated themselves as less happy (42).

The present study argued the 100-point numerical rating scale is better than other scales to measure self-rated health and happiness. First, scores can be obtained in written or oral form, and it is simple to administer and score. It is reasonable for older adults who might be illiterate or have vision or hearing problems. In contrast, the visual analog scale can be administered only in writing. Second, regarding the psychometric criteria of reliability and predictive validity, there is the advantage of having 101 response options (44–46), which is likely to appeal to researchers concerned with the limited response options offered by ordinal scales (7). Third, it has the advantage over ordinal scales of being able to assess criterion validity.

This study had several strengths. First, the sample size of participants was large. Second, face-to-face interviews at the participants' homes reduced information bias. Third, it was the first study to determine the corresponding cut-off scores on global self-rated health and global self-rated happiness relative to the PCS and MCS separately by gender and age group. However, it had some limitations. First, the sample's demographic characteristics differed from that of the registered elderly residents of Yilan city. However, because this study was not an epidemiological survey, the sociodemographic representativeness of this sample is not expected to compromise the generalizability of our findings. In contrast, the physical and cognitive demanding nature of our interview protocol suggests that the generalizability of our findings is limited to community-dwelling older adults with no serious physical and cognitive disability. The generalizability of our findings in additional populations such as those institutionalized and with severe disabilities should be further examined in the future. Second, it was not clear that the SF-12v2 was the most appropriate instrument for older people because health-related quality of life among older people might be focused on physical aspects at the expense of other quality-of-life dimensions (47). However, targeted measures have not yet been developed for older adults.

## Conclusion

This study's results suggest that global measures of self-rated health and self-rated happiness are valid instruments for quick assessments of the physical and mental health states of Chinese older adults, who reside in the community, remain socially active, and do not have any serious disability. Further, the cut-off scores we calculated to indicate optimal physical and mental health scores seemed to be age- and gender-specific, and the reasons for age and gender differences in global self-rated health and self-rated happiness among older adults should be investigated.

## Data Availability Statement

The datasets generated for this study are available on request to the corresponding author.

## Ethics Statement

The studies involving human participants were reviewed and approved by Institutional Review Board of National Yang-Ming University Hospital (IRB No. 2011A016). The patients/participants provided their written informed consent to participate in this study.

## Author Contributions

Y-HL and N-WH initiated the study. Y-HL, N-WH, and H-CC managed the data collection, performed the data analysis, and wrote the first draft of the manuscript. Y-HL, N-WH, H-CC, and PC are collectively responsible for interpreting the results and reviewed critically subsequent drafts of the manuscript. All authors contributed to its design read and approved the final manuscript.

## Conflict of Interest

The authors declare that the research was conducted in the absence of any commercial or financial relationships that could be construed as a potential conflict of interest.
