# Supplementary material for: Validation of Global Self-Rated Health and Happiness Measures Among Older People in the Yilan Study, Taiwan
Source: Front Public Health. 2020 Jul 31;8:346. doi: 10.3389/fpubh.2020.00346 (PMC7411153; doi:10.3389/fpubh.2020.00346)
Supplement: Supplementary file 1 [file Table_1.DOCX]

Table S1. Optimal cut-off scores and validity results on global self-rated health (relative to the PCS) and global self-rated happiness (relative to the MCS) cut-off scores of 50; *n* = 3,982.

|  | **Physical Component Summary** | | | | **Mental Component Summary** | | | |
| --- | --- | --- | --- | --- | --- | --- | --- | --- |
| **Group** | **Optimal^a^** | **Sensitivity** | **Specificity** | **Area under curve** | **Optimal^b^** | **Sensitivity** | **Specificity** | **Area under curve** |
| Total | 68.5 | 0.74 | 0.62 | 0.74*** | 69.5 | 0.74 | 0.60 | 0.72*** |
| Gender | |  |  |  |  |  |  |  |
| Male | 68.5 | 0.76 | 0.61 | 0.75*** | 69.5 | 0.76 | 0.60 | 0.74*** |
| Female | 67.0 | 0.73 | 0.63 | 0.74*** | 62.5 | 0.77 | 0.57 | 0.71*** |
| Age (in years) | |  |  |  |  |  |  |  |
| 65–74 | 67.0 | 0.73 | 0.61 | 0.73*** | 69.5 | 0.77 | 0.59 | 0.73*** |
| 75+ | 69.0 | 0.77 | 0.63 | 0.76*** | 62.5 | 0.76 | 0.57 | 0.71*** |

* = *p* < 0.001.

^a^Optimal cut-off scores on global self-rated health.

^b^Optimal cut-off scores on global self-rated happiness.
